# Zoonotic filariasis and its public health significance: a comprehensive literature review

**DOI:** 10.3389/fmicb.2025.1700645

**Published:** 2025-11-14

**Authors:** Remya M., Manju Rahi, Prasanta Saini

**Affiliations:** ICMR-Vector Control Research Centre, Puducherry, India

**Keywords:** filariasis, zoonosis, setariasis, dirofilariasis, onchocerciasis

## Abstract

Animal filariasis, a group of vector-borne parasitic infections, is a widely significant yet often underreported complex disease affecting a broad range of domestic and wild animals across tropical and subtropical regions. This comprehensive literature review aims to compile current knowledge on its epidemiology, pathogenesis, diagnosis, economic impact, and zoonotic implications to support improved control strategies. This review was conducted using databases such as PubMed, Scopus, and Google Scholar to collect data on filarial species, their vectors, hosts, clinical manifestations, diagnostic methods, and control measures across different geographical regions. Disease is caused by filarial nematodes belonging to order Spirurida, family Onchocercidae, which include species such as *Setaria, Dirofilaria, Onchocerca, Stephanofilaria, and Parafilaria*. This disease transmission primarily depends on blood sucking arthropod vectors, such as mosquitoes, blackflies, and biting midges. Distribution and involvement of these vectors influenced by environmental conditions, host availability, and regional factors. The lifecycle of all members of Onchocercidae family uniformly exhibit a similar pattern. Clinical manifestations vary depending on the host and parasites, can cause a mild to severe conditions including peritonitis, dermatitis as skin nodules, ocular infections, neurological disorders and cardiopulmonary complications. Worms have been detected in various tissues, including subcutaneous tissues, lymphatics, eye, heart, lungs, and central nervous system. Zoonotic infections have involved species from genera such as *Setaria*, *Dirofilaria*, *Onchocerca*, *Brugia*, *Dipetalonema*, *Loaina*, and *Meningonema*. Diagnosis of filarial infections relies on conventional methods like blood smears and skin scrapings, along with advanced molecular and serological assays for enhanced sensitivity and species identification. Control strategies include routine prophylactic treatment, vector control, and increased awareness among livestock farmers and pet owners. Filariasis in livestock causes economic losses through reduced productivity, fertility, hide quality, and treatment costs and meat condemnation. It also leads to higher management expenses, trade barriers, and decreased agricultural efficiency, particularly in endemic regions. A comprehensive understanding of intricate interactions among filarial parasites, mosquito vectors and vertebrate hosts is crucial for formulating effective prevention and control strategies. So, integrated control programs and one health approach, driven by interdisciplinary research and public health collaboration, are crucial to addressing this challenge and mitigating its zoonotic potential.

## Graphical abstract

**Figure:**
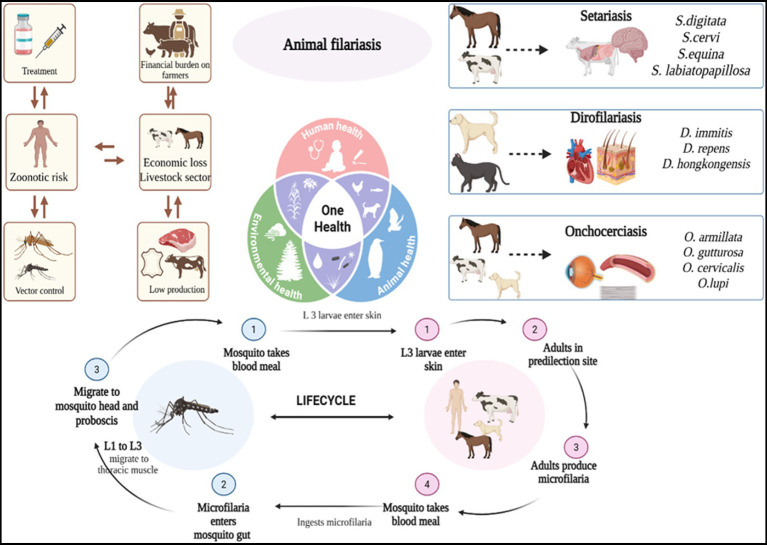

## Introduction

Filariasis is an arthropod-borne zoonotic disease caused by various species of filarial nematodes, transmitted by the bites of infected blood-sucking arthropods such as mosquitoes, ticks, sandflies, and black flies. Mosquito belonging to various genera, like *Aedes, Anopheles, Armigeres, Culex, Mansonia* and *Ochlerotatus* are involved in the transmission of filariasis (Orihel and Eberhard, 1998; Foster and Walker, 2019). Mainly, *Culex, Aedes* and *Anopheles* are primary carriers for Dirofilariasis and Setariasis. Black flies (*Simulium* spp.) are known to transmit Onchocerciasis, especially in livestock and wildlife. Biting midges (Culicoides) contribute to transmission of *Onchocerca*, *Setaria* and *Stephanofilaria* species in cattle. Additionally, horseflies (*Tabanus* spp.) can serve as mechanical or biological vectors for *Parafilaria* and some *Setaria* species. Therefore, tropical and subtropical areas are where these diseases are most common. It is essential to comprehend the complex interactions among filarial parasites, vertebrate hosts, and mosquito vectors in order to evaluate the risks of disease transmission and create efficient preventative and control measures (Siriyasatien et al., 2023).

The order Spirurida, superfamily Filarioidea comprises relatively long and thin worms, primarily belonging to three families: Filariidae, Setariidae, and Onchocercidae (Soulsby, 1982). Among these *Dirofilaria* spp., *Setaria* spp., *Onchocerca* spp. pose a major threat to both animals as well as humans, while *Stephanofilaria* spp. and *Parabovicola* spp. have been reported to cause minor infections in animals. In their definitive vertebrate hosts, filarial nematodes generate moving microfilariae (mf) to be their first-stage larvae (L1). Microfilarial larvae, enclosed in a thin, flexible membrane, enter the bloodstream or lymphatic tissue of host. From there, they are ingested by mosquitoes, which serve as intermediate hosts and biological vectors. These microfilariae then develop into third-stage larvae (L3) within blood-feeding arthropods (Orihel and Eberhard, 1998; Otranto and Deplazes, 2019). In blood-feeding arthropods, these microfilariae subsequently mature into third-stage larvae (L3) (Otranto and Deplazes, 2019). When the mosquito feeds on a new host, L3 larvae proceed to proboscis inside the mosquito and escape. The microfilariae then complete their development within the final host. Notably, microfilariae can survive in the host for several weeks after the death of the adult worm, and can also be transmitted to uninfected hosts (Soulsby, 1982). Animal filariasis exhibits different predilection sites different hosts, affecting various body systems, including heart, lungs, subcutaneous tissues, eyes, lymphatic system, and central nervous system. Adult worms typically reside in connective tissues, lymphatic channels, internal cavities, and blood vessels of host animals (Chatterjee and Nutman, 2015). Moreover, filarial parasites are found in various vertebrates, but only those in mammals can be transmitted to humans, posing a zoonotic risk (Anderson, 2000). So, the symptoms of filariasis vary depending on the location of adult and larval nematodes, with infection severity linked to parasite species and abundance (Magnis et al., 2013). Although these infections are widespread across tropical and subtropical regions, they remain neglected and underreported in veterinary and public health sectors. The disease causes substantial economic losses through reduced animal productivity, reproductive failure, and increased mortality, while zoonotic transmission continues to emerge as a growing health concern.

Zoonotic filariasis, caused by animal derived filarial infections in humans, is distributed world-wide. First report in modern literature over a century ago, both in number of cases and number of implicated parasitic species have steadily increased. So, recently, a wide range of filarial species pose a significant risk to humans as well. Although, Humans are mainly affected by lymphatic filariasis such as *Wuchereria bancrofti* as well as to a lower degree, *Brugia malayi* and *Brugia timori*, which seriously impair the health of almost 68 million people in 73 countries. In humans, a number of filarial parasites have been found, including *Wuchereria, Brugia, Dirofilaria, Onchocerca, Dipetalonema, Loaina*, and *Meningonema* (Ramaiah and Ottesen, 2014). Animal filariasis also poses a significant threat to human health, notably through onchocerciasis caused by *Onchocerca volvulus*, parasite responsible for river blindness. This disease affects approximately 40 million humans, primarily from Africa (Taylor et al., 2010; Tekle et al., 2016). Another form of animal filariasis, particularly that caused by *Dirofilaria repens*, is more common in males and children (Pampiglione et al., 1995). Epidemiology of these infections is primarily impacted by human activity and environmental factors associated with parasite vectors; incidence rates are higher in regions with longer mosquito breeding seasons and higher levels of outdoor exposure, whether for work or pleasure. So, close contact between humans and animals heightens the risk of zoonotic disease transmission and newly emerging zoonotic diseases represent a major threat to public health and significantly disrupts socioeconomic stability (McArthur, 2019). Several methods are typically used to diagnose filarial infections, such as separating adult worms and then identifying them morphologically, observing circulating microfilariae morphologically using direct wet smears, stained blood smears, modified Knott’s technique, and Wylie’s filtration technique (Irwin and Jefferies, 2004). Recombinant antigen-based assays and molecular techniques enhance diagnostic sensitivity and specificity while also increasing diagnostic accuracy. Additionally, histochemical or immuno-histochemical staining (IHC) of blood microfilariae has been used as a diagnostic technique detecting filarial infections (Ananda et al., 2006). Numerous genes have been described according to their potential for therapeutic targeting, including their protein products. Moreover, molecular diagnostic techniques are being used more and more for surveillance and research (Casiraghi et al., 2006; Rishniw et al., 2006). Although wide-spectrum anthelmintics are frequently used to treat and manage filarial infections, there remains a pressing need for new antifilarial therapies that can specifically target and eliminate adult parasites (Hoerauf, 2008). Despite being one of the most serious issues facing public health world-wide, resulting in fatalities in livestock, humans and wildlife, as well as significant economic losses, vectors pose direct impacts such as bites, irritation, psychological stress, and discomfort during feeding. Additionally, they serve as carriers of various diseases and incur substantial costs for their control. Implementing vector control through the modern approach of integrated Pest management offers the most effective solution, as it not only enhances control efficiency but also helps mitigate issues related to acaricide resistance and reduces the environmental risks posed by harmful chemical use. Furthermore, these disease affects the productivity, health, and reproductive performance of infected animals, resulting in reduced milk yield, weight loss, poor growth rates, decreased work capacity, and increased mortality. These outcomes directly impact livestock producers, industries, and national economies. Indirectly, the presence of the disease may necessitate changes in farming practices and labour allocation, ultimately reducing household income and food security (Perumal et al., 2016). Despite decades of research on human lymphatic filariasis, the epidemiological patterns, host–vector relationships, and cross-species transmission dynamics of animal-derived filarial infections are poorly understood. Most available data are fragmented, region-specific, and lack integration under a One Health framework. Furthermore, limited molecular and surveillance studies constrain our ability to monitor emerging zoonotic strains and design evidence-based control measures. To address these gaps, this study investigates the increasing occurrence of zoonotic filarial infections in humans and animals, emphasizing their clinical manifestations, economic impact, and ecological determinants. Although several studies have explored the epidemiology and control of filariasis, limited attention has been given to the zoonotic potential of animal filarial infections and their role in sustaining transmission in endemic regions. By consolidating current evidence, the review aims to identify research gaps and inform integrated control strategies under a one health framework.

Therefore, insights gained from studies on the interactions between filarial parasites and mosquito vectors are crucial for assessing disease transmission risks and guiding effective mosquito control strategies in endemic regions. In the present study, we investigate the rise in humans and animal zoonotic filarial infections, emphasizing their potential to cause clinical symptoms such as peritonitis, dermatitis, ocular complications, skin nodules, cardiopulmonary diseases and epilepsy. These findings highlight the critical need for one health strategies to detect and manage the emerging infections effectively. The objective is to highlight the current knowledge by conducting a thorough examination of the economic impact of zoonotic diseases on different species, adopting a one Health approach that highlights the intricate relationships between human, animal, and environmental wellbeing.

## Materials and methods

A comprehensive literature review was conducted to explore the zoonotic filariasis and its public health significance mainly dealing seteriasis, dirofilariasis and onchocerciasis. This review synthesizes current knowledge on the epidemiology, pathogenesis, diagnosis, economic impact, and zoonotic implications of animal filariasis. We conducted a database search of PubMed, Scopus, Web of Science, and Google Scholar, retrieving articles from their inception through April 2025. The search keywords included animal filariasis, zoonotic filariasis, seteriasis, onchocerciasis, dirofilariasis, impacts of filariasis in livestock sector. The inclusion criteria consisted of English-language, peer-reviewed articles that offered cutting-edge perspectives on animal filariasis, encompassing epidemiology, pathogenesis, diagnosis, economic burden, and zoonotic significance mainly on seteriasis, dirofilariasis and onchocerciasis. The exclusion criteria included human lymphatic filariasis and animal filarial diseases other than setariasis, onchocerciasis, and dirofilariasis. Through critical analysis of selected articles, we examined the progression, technical aspects, challenges, and future directions of zoonotic animal filariasis. Our findings underscore the need for a One Health approach, integrating interdisciplinary research and public health partnerships to effectively control zoonotic risk.

## Animal filariasis

*Setaria, Dirofilaria,* and *Onchocerca* are key genera in animal filariasis, characterized by distinct epidemiological patterns and transmission dynamics. The disease cycle is vector-borne, involving arthropods such as mosquitoes, blackflies, and biting midges, which transmit infective larvae to vertebrate hosts. Beyond their impact on animal health and productivity, certain filarial species exhibit zoonotic potential, posing significant public health concerns. This section provides a comprehensive overview of these genera, focusing on their life cycles, pathology, and implications for public health.

## Setariasis

Setaria is a type of nematode that often lives free within peritoneal cavity of ungulates. It is a member of family Setariidae and order Spirurida, is transmitted by biting insects like flies (*Haematobia, Simulium*) and mosquitoes (*Aedes, Anopheles, Culex, and Mansonia*) (Soulsby, 1982; Azari-Hamidian et al., 2019). There are 43 known species of nematodes in the genus *Setaria*, that inhabit the abdominal cavity of artiodactyl animals, including cattle, sheep, horses, and pigs, with a global distribution as enlisted in Table 1 (Laaksonen et al., 2009). Three of these, *S. digitata*, *S. cervi* and *S. labitopapillosa* have been reported from India, six from Europe, and five from America. Adult worms (about 5 to 10 cm) reside freely within abdominal cavity, while their microfilaria (190 μm in length) circulate in blood of definitive host. Each setaria species has a specific host preference; for example, *S. equina* predominantly infects horses, while *S. digitata* and *S. labiatopapillosa* are found in ruminants (Davoodi, 2015). In addition, *S. cervi*, *S. equina S. tundra, S. marshalli, S. leichungwingi* and *S. nelsoni* are also found in cattle (Sundar and D’Souza, 2015). These parasites predominantly inhabit in peritoneal cavities, particularly in both domestic and wild animals (Gomez-Puerta and Mayor, 2017; Mrifag et al., 2021). In Asia, *S. digitata, S. labiatopapillosa*, and *S. marshalli* are the most common nematodes observed in cattle (Khedri et al., 2014). Moreover, *S. digitata* and *S. labiatopapillosa* parasites can accidently infect humans. Also, *S equina* originating from horses and donkeys, has been responsible for rare zoonotic transmissions, occasionally causing to subconjunctival eye infections (Nabie et al., 2017; Ţălu et al., 2012).

**Table 1:** Different species of *Setaria*, their predilection sites, and associated clinical signs.

**Species**	**Host**	**Predilection site**	**Clinical signs**	**References**
*S. digitata*	Cattle Buffalo	Peritoneal cavity	Fibrinous peritonitis (mild insignificant)	Bazargani et al. (2008), Tamilmahan et al. (2013), Davoodi (2015), Abdullah et al. (2021), Lee et al. (2021), and Hanafiah et al. (2023)
	Goat Sheep	CNS	Cerebrospinal nematoidosis Lumbar paralysis Death	
	Horse	CNS Eye	Equine neurological ataxia Lacrimation, Photophobia Corneal opacity Conjunctivitis Loss of vision	
	Human	Eye Lungs Urinary bladder Skin	Abscess formation Allergy responses Enlarged lymph node Lung inflammation Eye lesions, Eosinophilic granulomatous lesions (in urinary bladder)	
*S. labiatopapillosa*	Cattle	Peritoneal cavity	Fibrinous peritonitis (mild insignificant)	Sundar and D’Souza (2015)
	Sheep Goat Horses Human	Eye & CNS	Ocular pathology, Neurological diseases, Death	
*S. equina*	Horses, Cattle, Buffalo	Sub conjunctiva	Eye infection	Devi et al. (2020)
*S. cervi*	Cattle Buffalo Deer	Peritoneal cavity CNS	Peritonitis Nervous lesions	Sundar and D’Souza (2015).
*S. marshalli*	Cattle Buffalo	Peritoneal cavity	Peritonitis	Tung et al. (2003)
*S. tundra*	Reindeer, Cattle and buffalo	Peritoneal cavity	Peritonitis	Lanková et al. (2021)

Setariasis, also referred to as setariosis, is a disease brought on by *Setaria* nematodes. In tropical areas where mosquito vectors are common, setariasis poses a serious risk to vulnerable animals. The life cycle begins when mosquitoes ingest microfilariae while mosquitoes feeding on the blood of infected host. After which, in 2 to 3 weeks, the mosquitoes mature into infectious larvae (L3). Additionally, L3 larvae are spread by infected mosquitoes to other hosts during subsequent blood meals, where they develop into adult nematodes over a period of 8–10 months (Perumal et al., 2016). Among the various species, *S. digitata* is the most widely recognized and is prevalent across Asia, with several reports of its occurrence in India (Chaithra et al., 2024). It is particularly notable for being a common cause of cerebrospinal nematodiasis (CSN) or neurological ataxia in equines (Lee et al., 2021). When *S. digitata* infections occur in its usual hosts, for example cattle and buffaloes, they often only result in moderate fibrinous peritonitis and are generally benign. However, immature worms migrate to odd places, including the central nervous system, when infectious L3 larvae are unintentionally transferred to aberrant hosts like goats, sheep, or horses, which may result in severe neurological consequences (Bazargani et al., 2008). Typical symptoms in infected goats often exhibit paralysis of one or both forelimbs or hind limbs, lumbar region paralysis, incoordination, and a swaying gait, which can progress to death (Bazargani et al., 2008). In horses, primary migration of *Setaria* infection often involves ocular migration, where the parasites migrate to either single/both eyes (Radwan et al., 2016). Infected horses may exhibit various ocular symptoms, including blindness, conjunctivitis, corneal opacity, excessive lacrimation, and photophobia, especially if treatment is postponed (Tamilmahan et al., 2013). In addition to animal infections, zoonotic cases of *S. digitata* have been reported in humans, resulting in abscess formation, allergic reactions, lymphadenopathy, ocular lesions, and pulmonary inflammation (Rodrigo et al., 2014). It may also lead to eosinophilic granulomatous lesions in the urinary bladder. Given these findings, *S. digitata* should be regarded as a significant health threat to animals, with serious economic consequences for livestock owners. Therefore, further epidemiological studies are essential, particularly focusing on both natural and aberrant hosts (Yu et al., 2021). *Setaria equina* is present in all part of the world and commonly seen in equines. This worm is found in the peritoneal cavity and sometimes in the scrotum. It has also been recorded from the pleural cavity and lung of horses and from the eye of horse and cattle. The infection rate is high and 50% horses are affected in endemic areas. *S. equina* is found not only in the peritoneal cavity but also in the scrotum, pleural cavity, lungs, and eyes of horses (Devi et al., 2020).

**Figure 1:**
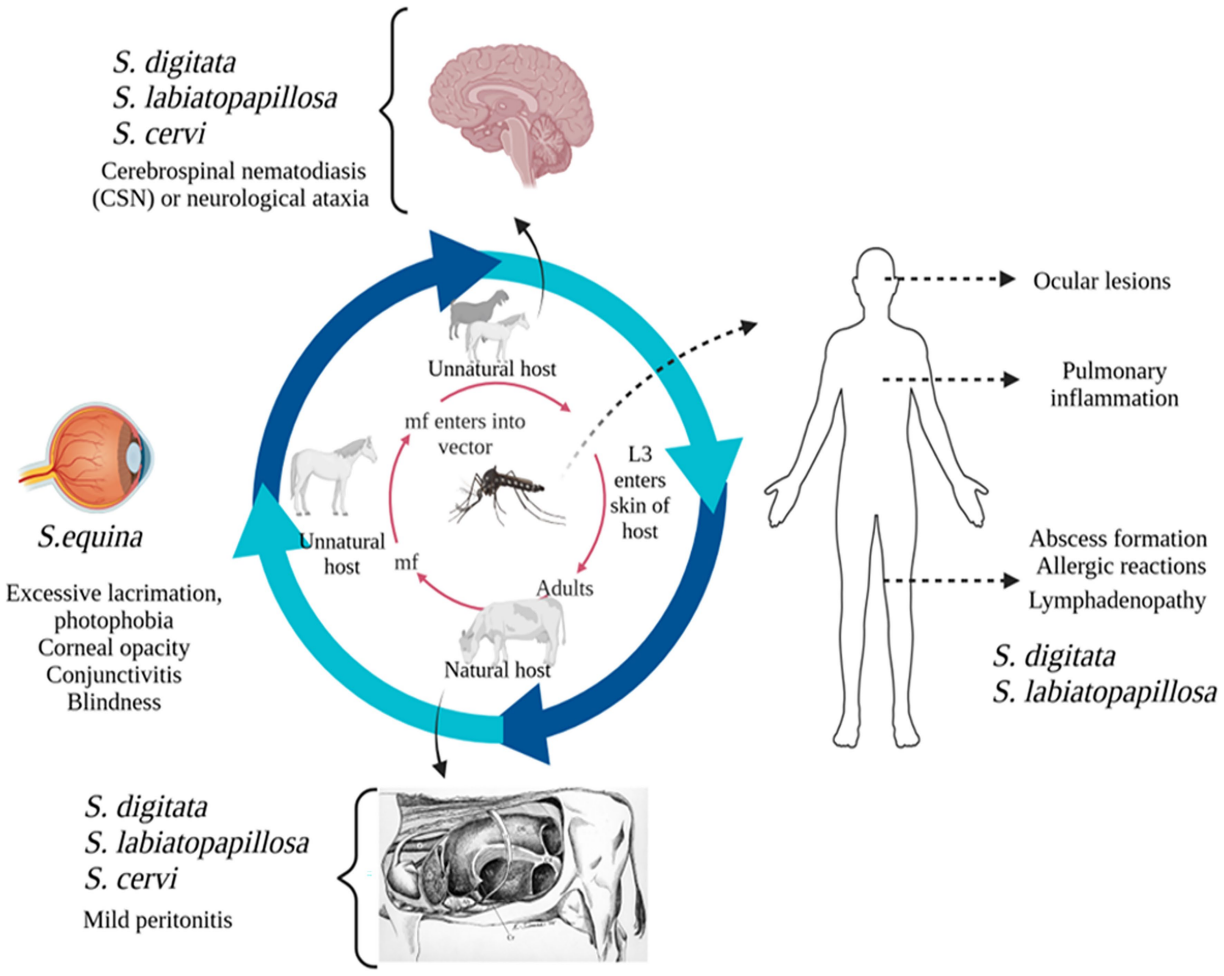
Diagrammatic representation of *Setaria* species and their predilection sites in natural and unnatural hosts.

The main pathogenic effects of *Setaria* spp. is the induction of various degrees of fibrinous peritonitis, mostly as a result of discomfort from mature worms living in the peritoneal cavity (Rhee et al., 1994). Although mature *S. digitata* parasites may persist in their typical host for as long as 18 months without suffering significant harm, migrating larvae can often invade other organs and produce pathological lesions. Nevertheless, they may have detrimental effects in unexpected places, like the central nervous system, nerves, eyes, and even the foetus of pregnant animals, which might cause serious impairment, paralysis, and death in a matter of days. In aberrant hosts, the main pathological conditions included ocular setariasis, cerebrospinal nematodiasis, and lumbar paralysis, this may result in blindness, neurological conditions, paralysis, corneal opacity, and eventually death (Tamilmahan et al., 2013; Abdullah et al., 2021). However, these parasites exhibit unpredictable migration patterns, often affecting the eyes and sometimes other organs like the CNS, urinary bladder, and reproductive system, as well as the liver, heart, and fetus in pregnant hosts (Yu et al., 2021). Several studies have reported that this filarial parasite can infiltrate horses’ and cattle’s eyes and cause blindness (Tamilmahan et al., 2013). Congenital infection has also been reported in animals, where the parasite penetrates the placenta, migrates to the fetus, enters the foetal bloodstream, and develops into adult worms (Kim et al., 2002; Schuster et al., 2019). Additionally, instances in cattle have been reported symptoms such as eye swelling, corneal damages, corneal cloudiness, and even blindness (Hanafiah et al., 2023). In horses, ocular setariosis is marked by the migration of parasite within anterior chamber of eye, often resulting in varying degrees of corneal opacity, including excessive lacrimation, photophobia, conjunctivitis and, in severe cases, partial or complete vision loss (Rafee and Amarpal, 2016). Interestingly, this parasite has been observed in multiple organs, including heart, lungs, spleen, kidneys, uterus, oviduct, ovaries, and urinary bladder. Also, it has been detected in the contents of the rumen, reticulum, and abomasum (Mandal and Ray, 1994). The effects of host–parasite interactions and predilections are shown in Figure 1.

The morphological identification of *Setaria* species, including *S. digitata*, *S. equina*, *S. labiatopapillosa*, and *S. cervi*, is often challenging due to their close morphological similarities (Maharana et al., 2020). To overcome this limitation, a nucleic acid-based detection approach was employed. Gel electrophoresis revealed a distinct 209 bp amplicon, and BLAST analysis of the corresponding genetic sequence confirmed the species as *S. digitata*, showing 99% similarity to isolates previously reported from cattle and buffaloes in Sri Lanka and China. This molecular identification was further substantiated by phylogenetic analysis, which clustered the isolate within the *S. digitata* clade (Yu et al., 2021).

## Dirofilariasis

Dirofilariasis is a zoonotic helminthic disease caused by *Dirofilaria* species, transmitted through mosquito vectors, and has a worldwide distribution, impacting both human and animal health. As summarized in Table 2, the disease predominantly affects domestic dogs, cats, and various wild mammals, highlighting its significance in both veterinary and public health contexts (Day, 2011; McCall et al., 2008). Infected animals commonly exhibit signs associated with cardiopulmonary system, in addition to subcutaneous, ocular, and dermatological symptoms. The adult worms primarily inhabit pulmonary arteries, where they release microfilariae into bloodstream. Mosquitoes, such as *Culex pipiens*, *Aedes albopictus*, *Anopheles maculipennis*, and *Coquilletidia richiardii*, acquire these microfilariae while feeding. After developing into infectious L3 larvae within the mosquito’s hemocoel, the larvae go to the mouthparts of the mosquito. After being spread by a mosquito bite, L3 larvae move to the pulmonary arteries and develop into adult worms. In mammalian host, larvae progress from L3 to L4 within somatic tissues over 3–12 days, then travel to the pulmonary arteries and right ventricle. In these locations, they undergo a final moult to L5 immature adults, which later develop into mature heartworms (Hoch and Strickland, 2008). In contrast to *D. immitis*, L4 larvae of *D. repens* do not migrate but moult and mature into an adult within the subcutaneous tissues.

**Table 2:** Different species of *Dirofilaria*, their predilection sites, and associated clinical signs.

**Species**	**Host**	**Predilection site**	**Clinical signs**	**References**
*D. immitis*	Dog Cat & wild felids Ferrets Monkeys	Pulmonary arteries Right ventricle	Right ventricular hypertrophy Pulmonary lesions (coughing, persistent expiratory lung sounds)	Day (2011), McCall et al. (2008), and Sevimli et al. (2007)
	Human	Pulmonary artery	Pulmonary nodules	
*D. repens*	Dog Cat	Subcutaneous tissue Subconjunctiva	Subcutaneous nodules	Petry et al. (2015), Genchi et al. (2010), and Albanese et al. (2013)
	Human	Subcutaneous tissue Subconjunctiva orbital zone Eyelids Intra vitreous tissues	Subcutaneous nodules Ocular lesions	
*D. hongkongnesis*	Dog Cat	Subcutaneous tissue	Subcutaneous nodules	Kwok et al. (2016), Luk et al. (2021), and Manathunga et al. (2024)
	Humans	Subcutaneous tissue Subconjunctiva	Subcutaneous nodules Ocular lesions	

In dog populations, both *D. immitis and D. repens* are common. However, *D. repens* poses a more substantial threat to humans due to its greater ability to survive and persist in both hosts and vectors compared to *D. immitis*. *D. immitis* heartworm infections are frequently observed in dogs between the ages of 5 and 6 years, with a higher incidence in male dogs. Additionally, cases of subcutaneous dirofilariasis have been documented in dog populations in India (Song et al., 2003; Traversa et al., 2010). Although other animals, such as domestic cats, ferrets, monkeys, and wild canids, are also prone to *D. immitis* infection, the associated risk factors for these species have not been extensively investigated (Venco et al., 2015; Bowman et al., 2016; Alsarraf et al., 2023). Also, Infection caused by *D. repens* are more commonly reported in males and children (Simón et al., 2012). Moreover, *Dirofilaria hongkongensis*, initially reported in China, is a direct cause of dirofilariasis in human beings and dogs (To et al., 2012). A rigid genetic connection among *D. hongkongensis* and other *Dirofilaria* species that infect humans and animals, including dogs and jackals, is revealed by phylogenetic analysis, which may indicate that their clinical presentations are comparable (Saini et al., 2024). Furthermore, various wildlife-associated *Dirofilaria* species are also reported, such as *D. ursi, D. striata, D. tenuis, D. spectans*, and *D. magnilarvata* have been implicated in dirofilariasis cases of humans (Simón et al., 2022; Perles et al., 2024). Many hosts, such as foxes, dogs, cats, and other mammals, get infected by these parasites.

*D. immitis*, a zoonotic filarial nematode, generally resides in the pulmonary arteries and, in severe cases, may extend to the right ventricle of the heart, leading to significant cardiovascular pathology in dogs and other canids. It can cause significant pathology in multiple organs, including the lungs, heart, liver, and kidneys, with the most severe changes observed in the pulmonary arteries, right ventricle, and kidneys. Clinical signs progress from mild symptoms such as persistent cough and elevated body temperature to more severe manifestations, including dyspnea, generalized weakness, ascites due to right ventricular failure, and potentially life-threatening complications such as cachexia, respiratory distress, and sudden death. Severe chronic pulmonary arterial disease caused by *D. immitis* infection can result in right ventricular hypertrophy due to increased pressure load on the heart. Electrocardiographic abnormalities, such as P pulmonale, are frequently observed, and various arrhythmias, including atrial premature beats and atrial fibrillation, may develop as a consequence of the disease’s impact on cardiac function (Sevimli et al., 2007). The presence of persistent expiratory lung sounds, even in the absence of coughing, is a potential indicator of heartworm disease. Sometimes laboratory findings on complete blood count (CBC) may include eosinophilia, thrombocytopenia, and neutrophilia, which should prompt consideration of *D. immitis* infection in their definitive host (Bendas et al., 2022).

In most canine cases of *D. repens* infection, condition is subclinical or presents with nonspecific symptoms, resulting in many infections going undiagnosed. Through the muscular connective fascia and subcutaneous tissue, the infectious larvae develop into adults and settle down there permanently. Interestingly, *D. repens* does not initiate an inflammatory response or form a connective tissue capsule around the living parasite, which can be seen moving actively beneath connective serous layers. As a result, the infection often goes unnoticed due to the lack of clear clinical signs (Petry et al., 2015; Genchi et al., 2010). Occasionally, skin conditions such as itching, conjunctivitis, eye irritation, swelling, and parasite-containing subcutaneous nodules are all noticeable (Albanese et al., 2013). Infections with *D. hongkongensis* have also been documented to produce subcutaneous nodules resembling those produced by *D. repens* (Manathunga et al., 2024). According to Tarello (2011), the subcutaneous nodules caused by *D. hongkongensis* are predominantly located in the posterior body regions, such as the scrotal and mammary areas, similar to the anatomical distribution of *D. repens* lesions. Approximately 85% of *D. repens-*related dermatological lesions are observed in the lumbosacral region, hind limbs, and perianal area (Tarello, 2011).

**Figure 2:**
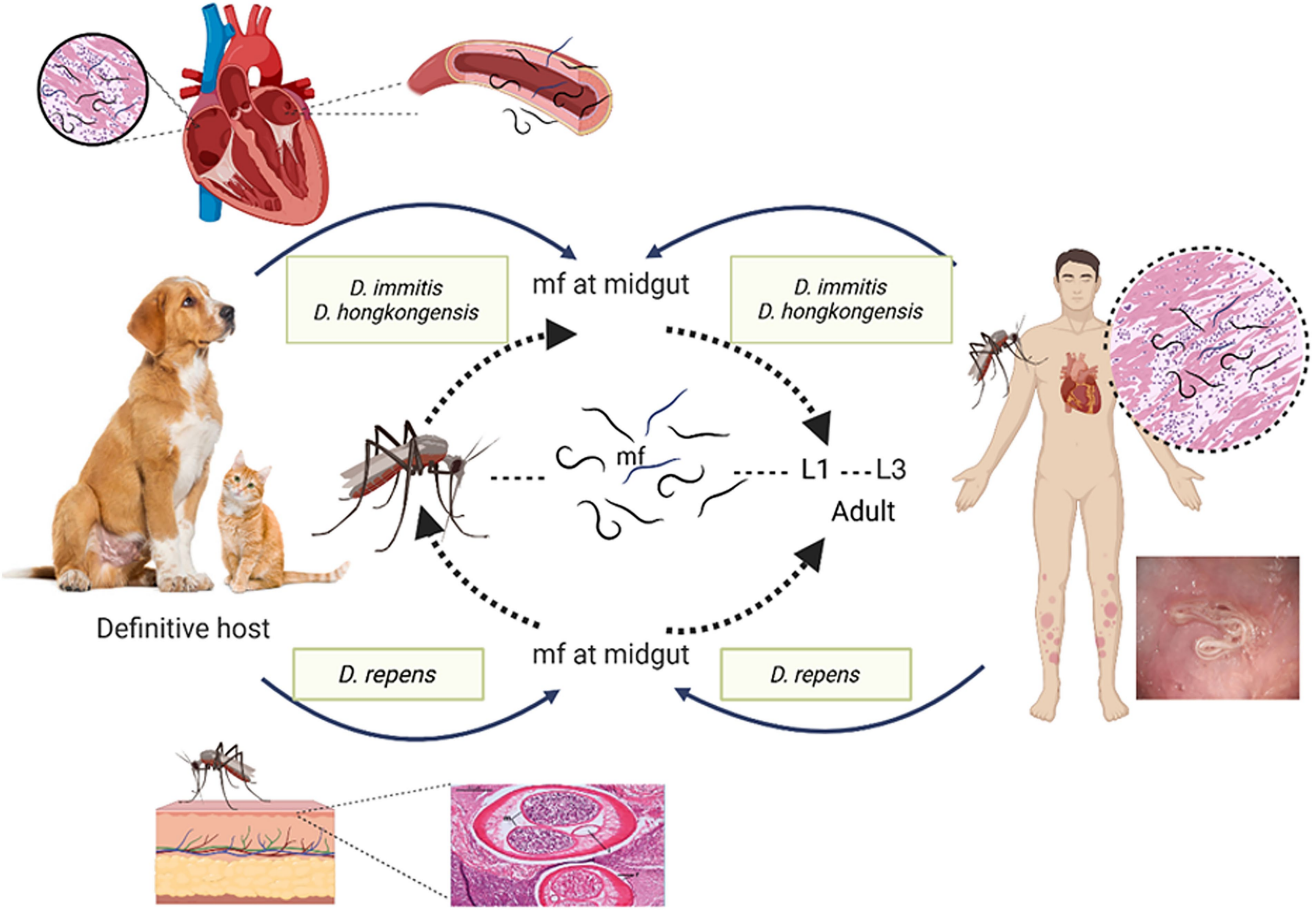
Diagrammatic representation of *Dirofilaria* species and different host range and clinical symptoms.

Canine dirofilariasis is a neglected zoonotic disease with significant implications for human health, as accidental infection can lead to pulmonary, subcutaneous, and ocular manifestations, potentially mimicking more severe conditions such as malignancies. Humans serve as incidental hosts for *D. repens* and *D. immitis*. Despite the rarity of human infections, *D. immitis* is usually linked to pulmonary lesions, which frequently appear as radiological coin type lesions in lungs (Foroulis et al., 2005; Theis, 2005). Individuals residing in endemic regions are at increased risk of infection through mosquito vectors, which facilitate the development of the parasite in subcutaneous tissues, mucosal membranes, and the subconjunctival space near the site of the mosquito bite. Additionally, some cases have reported the presence of tumor-like lesions in the lungs (Leccia et al., 2012). Rare instances of *D. immitis* and *D. repens* larvae have been documented in atypical sites, including mesentery, eye, spermatic cord, cerebral arteries and liver (Theis, 2005; Kim et al., 2002). Similarly, *D. hongkongensis* has also been identified in ocular (Kwok et al., 2016) and subcutaneous forms (Luk et al., 2021) in humans as illustrated in Figure 2. Given zoonotic nature of dirofilariasis and its significant clinical implications underscore the importance of early diagnosis, prevention through vector control measures, and targeted treatment strategies to mitigate the disease’s impact on both veterinary and human health. For the disease to be controlled and prevented, as related to other vector-borne infections, precise understanding of natural reservoir and vectors is essential.

Diagnosis of dirofilariasis relies on a combination of traditional and modern diagnostic techniques aimed at detecting the parasite in both humans and animals. Microscopic methods, such as Knott’s test and blood smear examinations using Giemsa or hematoxylin and eosin (H&E) stains, are employed to identify microfilariae in blood samples. Serological assays, including ELISA, indirect fluorescent antibody (IFA) tests, and immunochromatographic tests (ICT), are used to detect specific antibodies or antigens associated with *Dirofilaria* infection. Radiographic and imaging techniques, such as chest X-rays and ultrasonography, help visualize adult worms, particularly in pulmonary or subcutaneous lesions. In certain cases, necropsy and histopathological examination of tissues allow for definitive identification of adult parasites. This integrated, multi-modal diagnostic approach enhances the accuracy and reliability of dirofilariasis detection (Trancoso et al., 2020; Pękacz et al., 2022). Molecular diagnosis of dirofilariasis primarily employs the polymerase chain reaction (PCR), a highly sensitive and specific technique for detecting *Dirofilaria* DNA in clinical and experimental samples. By amplifying species-specific gene sequences, PCR provides superior accuracy compared to conventional diagnostic methods (Obradovic et al., 2013). Commonly targeted genes include the internal transcribed spacer (ITS) regions, cytochrome c oxidase subunit 1 (cox1), and 12S ribosomal RNA (12S rRNA), which facilitate precise species identification and phylogenetic analysis. In addition, advanced molecular platforms such as next-generation sequencing (NGS), loop-mediated isothermal amplification (LAMP), digital PCR (dPCR), and digital microfluidics have been increasingly applied. These technologies integrate molecular amplification with high-resolution imaging and computational analysis, offering rapid, reliable, and quantitative detection of *Dirofilaria* species (Aththanayaka et al., 2024). These advancements collectively strengthen diagnostic and healthcare practices, underscoring the importance of continued collaboration in research, standardization of diagnostic protocols, and effective implementation strategies. Such integrated efforts are essential to improving the accuracy of detection, guiding timely interventions, and ultimately enhancing the overall management and control of dirofilariasis.

## Onchocerciasis

There are around 30 species in the genus *Onchocerca*, which primarily parasitic to ungulates and are widely distributed throughout the world (Lefoulon et al., 2017). Moreover, *Onchocerca* spp. exhibits a highly specific host range (Rommel and Boch, 2000), with strong affinity for particular ungulates like bovines and equines as described in Table 3. Besides, these are also reported in canids, felids, and humans. Although primarily zoonotic, certain Onchocerca species, notably *Onchocerca volvulus*, have been identified as significant human pathogens, causing onchocerciasis in humans (Lefoulon et al., 2017). Transmission of Onchocerca nematodes occurs via arthropod vectors, primarily blackflies of genera *Simulium* and, in some cases, *Culicoides*, which serve as intermediate hosts and facilitate its lifecycle (Taylor and Muller, 1979). The lifecycle of all *Onchocerca* species is indirect, involving a dual host system such as an intermediate host (arthropod vectors, such as blackflies) and a definitive host (vertebrates, including animals and humans), where the parasites undergo progressive maturation and development (Rommel and Boch, 2000). Adult Onchocerca parasites exhibit a prolonged lifespan, persisting for 10–15 years within the host, where they reside and reproduce within fibrous nodules known as onchocercomas. These nodules are predominantly located in subcutaneous tissues but can also be found in various connective tissues, including ligaments, tendons, perimysial sheaths, and cartilage (Mather and Treuting, 2012).

**Table 3:** Different species of *Onchocerca*, their predilection sites, and associated clinical signs.

**Species**	**Host**	**Predilection site**	**Clinical signs**	**References**
*O. armillata*	Cattle buffaloes, dromedaries, goats	tunica intima of the aorta	Acute aortitis Eosinophilic inflammation to chronic granulomatous, Calcifying and fibrosing aortitis,	Neary et al. (2010), Sazmand and Joachim (2017)
*O. gutturosa*	Cattle, camels, dromedaries, horses, humans	Cervical ligaments	Subcutaneous nodules	Bino Sundar et al. (2017) and and Cambra-Pellejà et al. (2020)
*O. volvulus*	Humans	Skin, Eye	Skin irritation Blindness	Lefoulon et al. (2017)
*O. cervicalis*	Horses, ponies, humans, donkeys	Skin Cervical ligaments	Skin nodules Impaired function of ligaments blindness	Dagnaw et al. (2016)
*O. lupi*	Wolf, Dogs, Cats, Humans	Occular tissues	Conjunctivitis, Exophthalmos, Periorbital swelling, Photophobia, Lacrimation,	Egyed et al. (2001), Sréter and Széll (2008), and Otranto et al. (2015)

Bovine onchocerciasis remains a relatively neglected disease, despite its widespread distribution in temperate and tropical regions largely due to its frequently asymptomatic or subclinical presentation, which can obscure its impact on cattle health and productivity (Rabeya Begam et al., 2015). In *O. armillata* infections in cattle, adult parasitic nodules are primarily located in the tunica intima of aorta, marking a unique predilection site compared to other *Onchocerca* species (Neary et al., 2010). In contrast to *O. armillata*, other onchocerca species infecting cattle, as *O. dukei*, *O. ochengi*, and *O. gutturosa*, exhibit a predilection for cervical ligaments, thoracic connective tissues and ventral connective tissues, highlighting species-specific tissue tropism (Neary et al., 2010; Sazmand and Joachim, 2017). *O. armillata* infections result in a range of pathological changes in the bovine aorta, from acute aortitis with necrosis and eosinophilic inflammation to chronic granulomatous, calcifying, and fibrosing aortitis, primarily affecting the media and adventitia, with potential intimal and endothelial damage and thrombosis (Mather and Treuting, 2012). *O. armillata* infections can lead to severe complications, including aneurysm formation and potential rupture due to aortitis. While adult worms may elicit a minimal host response, microfilariae can cause significant clinical disease through inflammatory reactions as they migrate and die in tissues (Mather and Treuting, 2012).

In horses, *Onchocerca* infections frequently present as asymptomatic subcutaneous nodules that may remain undetected by owners. Nevertheless, in some cases, these infections can progress to cause dermatitis, ligamentous dysfunction, and ocular complications, including blindness (Lia et al., 2017). Adult *Onchocerca* parasites exhibit a predilection for specific anatomical locations, primarily residing within the ligamentum nuchae of equine hosts, where they form characteristic coiled aggregates. Additionally, they may localize within flexor tendon and fetlock suspensory ligament connective tissues, to produce that affect tissue function (Cambra-Pellejà et al., 2020). Among these, *O. cervicalis* is a prevalent species within the onchocerca genus, primarily infecting equine hosts, including horses, donkeys, and mules, and contributing to the burden of onchocerciasis in these animals. In addition to *O. cervicalis*, other Onchocerca species, including *O. reticulata* and *O. raillieti*, have been identified in equine hosts, but with lower prevalence rates. Female filarial nematodes release large numbers of microfilariae, which localize in the dermal layer after migrating through connective tissues, with common sites including the ventral abdomen, thoracic region, withers, neck, and facial areas (Dagnaw et al., 2016).

In canines, the localization of *Onchocerca* species may occasionally resemble that observed in other hosts. However, a reported case of an *Onchocerca* nodule protruding into the tracheal lumen led to severe respiratory distress, including coughing, dyspnea, suffocation, and eventual death (Papaioannou et al., 2004). Canine onchocerciasis is regarded as an atypical infection in which *O. lienalis*, a bovine parasite, accidently infects a canine host and localizes in an ectopic site (Eberhard et al., 2000; Zarfoss et al., 2005). *O. lupi* is a parasitic nematode that predominantly infects dogs but can also affect cats (Otranto et al., 2015). In definitive hosts, adult *O. lupi* worms exhibit a specific tissue tropism, localizing within the connective tissues of ocular structures, including the subconjunctiva, conjunctiva, eyelids, and nictitating membrane, where they reside on the sclera until reaching sexual maturity (Egyed et al., 2001; Sréter and Széll, 2008). These worms are characteristically coiled and confined within distinct nodules in connective tissue. Occasionally, this exhibits aberrant migration to atypical sites, including the laryngeal soft tissues in dogs (Alho et al., 2016) and spinal cord in humans (Chen et al., 2015; Dudley et al., 2015). Both acute and chronic ocular diseases are possible manifestations of canine onchocerciasis. Clinical symptoms of acute infections include ocular discharge, pain, lacrimation, periorbital oedema, conjunctivitis, exophthalmos, and photophobia; these symptoms usually appear without development of granulomas or cysts surrounding adult parasites (Eberhard et al., 2000; Egyed et al., 2002).

The rising prevalence of *Onchocerca* species in both wildlife and domestic animals, alongside habitat expansion driven by climate change and urbanization, has been identified as an important factor in amplifying human-parasite interactions, thereby increasing the risk of zoonotic transmission (Uni et al., 2015). Zoonotic *Onchocerca* infections in humans, although rare, can lead to clinical manifestations such as dermatitis and ocular complications, with some cases also associated with epilepsy (Gumisiriza et al., 2020). Generally, prevalence of onchocerciasis is higher in adults than in juveniles due to the prolonged prepatent period of the infection (Boijsen et al., 2017). Among this, *O. volvulus* is the primary pathogenic species affecting humans, typically manifests as a pruritic skin disease and can also lead to a range of visual impairments, with untreated cases potentially progressing to irreversible blindness (Brattig et al., 2021). Adult *O. volvulus* worms are typically encapsulated within subcutaneous nodules under skin, whereas microfilariae exhibit a specific distribution pattern in the dermal tissues, predominantly localizing on the hips, shoulders, and lower body regions (Rodríguez-Pérez et al., 2011). Additionally, major public health threat of *O. volvulus* occurs in conjunction with its Wolbachia endosymbiont, is a causative agent of river blindness, a major cause of vision loss worldwide. Mass ivermectin administration effectively reduces microfilarial loads and pathology, but its inability to target adult worms, which can live for over a decade, poses a challenge for disease control.

**Figure 3:**
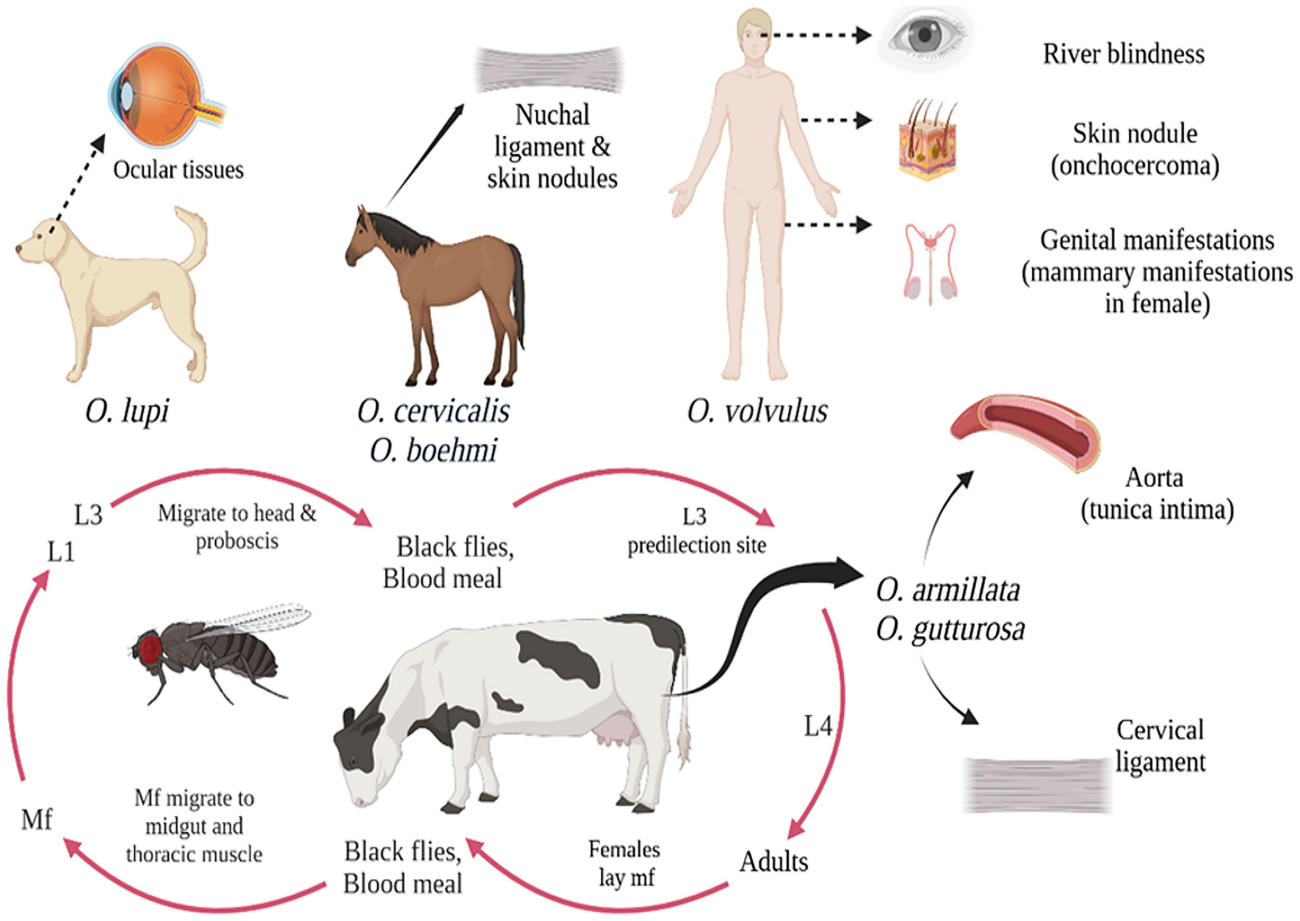
Diagramatic representation of *Onchocerca* species and different host range and clinical symptoms.

*Onchocerca* species exhibit host specificity, establishing patent infections primarily in closely related species, with microfilariae localizing in skin, subcutaneous tissues, and ocular structures in definitive hosts are shown in Figure 3. *Onchocerca* evolution suggests, co-speciation between parasites and hosts is not predominant mechanism for speciation. Rather, there is evidence that sympatric speciation mostly happens as a result of site movement and host switching (Morales-Hojas et al., 2006; Krueger et al., 2007). Typical host switching in *Onchocerca* is exemplified by species like *O. volvulus* (humans), *O. dewittei* (wild boars), and *O. fasciata* (camels), while site shift speciation is seen in *O. gutturosa* (cattle) and *O. lienalis* (cattle). *O. Lupi*, a parasite of dogs, illustrates a combination of both host switching and site shifting in its evolutionary history. So, understanding the mechanisms of speciation and host adaptation in *Onchocerca* is crucial for developing more effective control and management strategies for these parasitic infections.

Diagnosis of onchocerciasis remains challenging due to the inherent limitations of existing diagnostic methods. Although dermatological examination and nodule palpation are non-invasive and practical for field use, they often lack sufficient specificity and sensitivity. In contrast, invasive diagnostic approaches, including skin snip microscopy, quantitative PCR (qPCR), and loop-mediated isothermal amplification (LAMP), provide greater accuracy but require trained personnel and laboratory infrastructure, limiting their application in endemic settings (Hotterbeekx et al., 2020). Among molecular techniques, qPCR assays have demonstrated high sensitivity by targeting parasite-specific DNA sequences such as O-150, actin, and mitochondrial genes. Despite significant progress in global elimination programs, the final phase of onchocerciasis control demands diagnostics that are more specific, sensitive, and scalable. The development of innovative tools that can be integrated into local surveillance programs, accommodate coendemic infections, and align with WHO performance benchmarks is critical. With the deployment of robust diagnostic strategies and sustained global commitment, achieving the 2030 elimination goals remains an attainable target, underscoring the central role of diagnostics in this effort (Lubbers et al., 2025).

## Economic losses in livestock and pet sector

Vector-borne zoonotic diseases have a profound impact on global health and economies, contributing to considerable morbidity, mortality, and economic losses in human and animal populations. These diseases pose a major threat to livestock, resulting substantial financial burdens on producers and the agricultural sector, thereby impeding socio-economic development (Perumal et al., 2016). Filarial infections in livestock result in clinical manifestations such as lethargy, growth retardation, and decreased feed efficiency, ultimately compromising productivity and yields. Severe cases can lead to significant reduction in livestock population, directly affecting farm profitability. The economic impact is further exacerbated by diagnostic, treatment, and management costs, as well as increased management expenses, trade restrictions, and reduced labour efficiency, culminating in substantial economic losses for farmers and the agricultural sector.

Climate change is expected to impact the distribution, frequency, and severity of infectious diseases by altering the ecosystems of disease vectors. Rising temperatures can enable vectors to expand their range, potentially introducing diseases to new areas. This can affect the transmission of diseases like lymphatic filariasis and onchocerciasis, which are influenced by changes in mosquito and black fly populations. Climate-induced changes in landscape patterns, such as flooding and desertification, can also alter the distribution of disease vectors, impacting disease transmission dynamics. The growing threat of climate change to public health and the livestock sector necessitates timely and coordinated action to protect vulnerable populations and mitigate economic impacts.

In tropical areas, setariosis is a serious hazard to vulnerable animals. The widespread distribution and economic impact of *Setaria* spp. have established them as major parasitic concerns, particularly in atypical animal hosts. This disease contributes to substantial economic losses in livestock production by adversely affecting animal health, reducing productivity, and increasing mortality rates. In cattle, *S. digitata* is primarily non-pathogenic when localized in the peritoneal cavity. However, the migration of immature worms can lead to neurological disorders, peritonitis, tissue damage, and reproductive complications, negatively impacting overall health and productivity. In sheep and goats, cerebrospinal nematodiasis manifests as symptoms like incoordination, hindlimb paralysis, and recumbency, often resulting in death (Dehkordi et al., 2015). Additionally, ocular infections and organ damage further contribute to disease severity in affected animals. These health impacts lead to considerable economic losses in livestock industry, as they reduce milk and meat production, increase mortality rates, and impose financial burdens on the agricultural sector through diminished productivity and higher veterinary expenses. This filarial parasite has been documented to invade the ocular structures of horses, resulting in blindness (Shin et al., 2017), which contributes to significant financial losses equine industry because of its negative impacts on animal health, performance, and management expenses. Vision impairment compromises coordination and responsiveness, diminishing horse’s reliability for work and increasing the risk of accidents. Horses experiencing partial or complete blindness are less marketable, leading to a significant reduction in their commercial value. Additionally, the breeding potential of affected horses is adversely impacted, thereby reducing long-term economic gains for breeders.

Human cases of dirofilariasis have clearly been on the rise in recent years, most likely as a result of enhanced transmission from prolonged exposure periods, growing mosquito populations, and climate change (Darchenkova et al., 2009). *D. immitis* infection causes heartworm disease, which has serious clinical effects on dogs and financial ramifications for pet owners. The disease’s cost is determined by dividing the costs into three categories: opportunity cost of treatment, treatment cost, and preventative cost (heartworm medicine). As the condition spreads geographically, its prevalence rises in regions where it is already present, and pet care standards increase globally, heartworm expenditures are expected to rise in the future (Klug and Drake, 2022).

Dirofilariasis poses a significant threat to health and survival of pet animals while also negatively impacting their overall welfare and imposing both financial and emotional burdens on pet owners. This disease primarily targets the cardiovascular and respiratory systems, often resulting in pulmonary hypertension, heart failure, and potential multi-organ dysfunction (Darchenkova et al., 2009). Beyond its physiological effects, dirofilariasis greatly diminishes the overall health and standard of living of impacted animals. Affected pets frequently exhibit symptoms such as lethargy, respiratory distress, and decreased endurance, which restrict their ability to participate in normal activities, including exercise and playing.

Onchocerciasis is a debilitating filarial disease with profound socio-economic consequences, particularly in tropical regions where vector flies flourish, have a favourable environmental condition throughout the year. Recognized by the World Health Organization as a significant impediment to socio-economic development since the 1980s, the disease severely impacts productivity and disrupts social and reproductive aspects of life due to blindness and other debilitating complications. Onchocerciasis continues to pose a considerable burden in resource-poor settings, exacerbating physical disability and social stigma, which in turn diminish the quality of life for affected individuals and impact land productivity (Moya Alonso et al., 2009). In livestock, *Onchocerca* species, such as *O. gutturosa*, pose substantial economic challenges by compromising meat quality, rendering affected portions of cattle carcasses unsuitable for human consumption, and adversely affecting the meat export sector (Bino Sundar et al., 2017; Cambra-Pellejà et al., 2020). To mitigate these effects, increased research efforts are necessary, particularly in understanding the vectors responsible for transmission and their ecological dynamics, to facilitate the development of effective control strategies. Given the proven success and cost-effectiveness of existing control programs, prioritizing the development of innovative tools and strategies to reinforce these efforts is essential for achieving disease eradication. As filarial worm infections become increasingly prevalent due to climate change and rising mosquito populations, prioritizing awareness and preventive measures is crucial. Enhancing public education on disease prevention, making preventive treatments more accessible, and promoting routine veterinary check-ups can help reduce both the financial and emotional strain on the livestock and agricultural industries.

## Prevention and control up to this era and future aspects

Filarial nematode infections are a significant cause of morbidity in both animals and humans. Current control methods rely on routine injection of microfilaricidal medications, which target larval stages of parasite, which disrupt the lifecycle of parasite and help reduce clinical manifestations (Addiss and Mackenzie, 2004). However, there is a critical demand for novel antifilarial treatments that specifically target and eliminate adult parasites (Hoerauf, 2008). Existing treatments rely on broad-spectrum anthelmintics such as macrolactones, levamisole, and benzimidazoles, which target both adult worms and microfilariae, highlighting the need for more targeted and effective therapies.

Due to the strong predilection of *Setaria* species for ocular tissues in equines, surgical removal remains the preferred treatment over anthelmintic therapy. By taking this strategy, the infestation will be resolved quickly and there will not be any serious intraocular immune-mediated reactions related to dead parasite, which could cause problems like tissue necrosis, structural eye degeneration, elevated intraocular pressure, or even globe rupture. Although it has been demonstrated that pharmacological treatment with diethylcarbamazine (DEC) at 20 mg/kg intramuscular (IM) effectively clears microfilaremia, multiple doses are necessary, and the drug is ineffective against adult parasites living in ocular tissues (Peng et al., 2019). Topical ophthalmic treatments and anti-inflammatory therapy are also advised to reduce clinical symptoms and encourage healing. Ivermectin (300 μg/kg) administered subcutaneously once has been shown to be an effective treatment for *S. digitata* induced microfilariasis. Despite this, resolution of microfilaremia occurs only 7 days post-treatment, with adult eyeworms being eliminated 15 days post-treatment. Complete resolution of ocular symptoms has been observed approximately 90 days following treatment (Muhammad and Saqib, 2007). Given the detrimental effects of ocular *Setaria* infections on equine health and the associated economic burden on owners, preventive strategies are crucial.

Heartworm disease, caused by *D. immitis*, is a dangerous and potentially fatal aliments affecting both dogs and cats. The medical management of persistent heartworm infection is complex, expensive, and associated with significant risks. In order to eradicate symbiotic bacterium *Wolbachia*, which has been demonstrated to be essential to *D. immitis* survival and reproduction, the main method is to employ arsenical medication melarsomine, frequently followed by a month-long course of doxycycline. Currently, only drug registered for adulticidal therapy in canine heartworm infections is an arsenical drug (melarsomine). The standard protocol consists of two intramuscular injections of melarsomine (2.5 mg/kg) administered 24 h apart, achieving approximately 90% efficacy in eliminating adult worms. An alternative three-dose protocol, consisting of a single initial dose followed by two additional injections 30 days later (24 h apart), has demonstrated improved efficacy, eliminating up to 98% of adult worms. This staged approach reduces the overall parasite burden and lowers the risk of severe pulmonary thromboembolism (American Heartworm Society, 2012). Macrocyclic lactones (MLs), such as ivermectin, milbemycin, moxidectin, and selamectin, are widely used for heartworm prevention. While ivermectin has demonstrated partial adulticidal activity when administered at 6–12 mg/kg monthly for 16 months, it completely eradicate adult worms within 30 months (McCall et al., 2008). However, prolonged ML therapy is not recommended treatment due to the risk of continued disease progression, thromboembolism, and potential worsening of clinical signs (Venco et al., 2004). Doxycycline has emerged as a promising adjunct therapy due to its macrofilaricidal effects on *D. immitis*. The elimination of *Wolbachia* reduces inflammation and decreases microfilarial burden. A combination of doxycycline (10 mg/kg/day for discontinuous cycles) and ivermectin (6 μg/kg weekly) has been shown to accelerate microfilarial clearance and improve adulticidal efficacy compared to either drug alone (Bazzocchi et al., 2008). Historically, heartworm prevention relied on daily administration of diethylcarbamazine (DEC) during mosquito season. Prophylaxis has been transformed by the introduction of macrocyclic lactones, such as selamectin, moxidectin, milbemycin, and ivermectin. These agents interrupt larval development within the first 2 months post-infection, providing effective monthly or even less frequent administration schedules. The long-term use of available heartworm treatments has raised concerns about emerging drug resistance. Evidence of resistance to MLs has been reported in various veterinary parasitic diseases, emphasizing the need for novel therapeutic strategies (Noack et al., 2021).

The primary approach to treating and controlling onchocerciasis relies on ivermectin, which is highly effective against the microfilarial stage of *O. volvulus* but has limited impact on long-lived adult worms residing in tissues. With onchocerciasis now designated for elimination, there is a pressing need for new treatments, particularly those with macrofilaricidal properties (Gokool et al., 2024). As there is no available vaccine, the current strategy for preventing and eliminating onchocerciasis depends on mass drug administration (MDA) programs. These programs involve large-scale distribution of ivermectin, either once or twice a year, without requiring prior diagnosis or direct supervision by healthcare providers (WHO, 2023). Ivermectin, a macrocyclic lactone, acts exclusively on microfilariae by eliminating them and inhibiting the release of new larvae from female worm’s uterus, temporarily suppressing reproduction. This dual mechanism can reduce skin microfilarial loads by up to 99% within 2 months of treatment (Basáñez et al., 2008). To effectively reduce filarial transmission, treatment must continue throughout the reproductive lifespan of adult worms, which can survive for up to 15 years. However, absence of drugs capable of directly killing adult worms remains a major limitation to the success of these programs. Additionally, concerns are emerging regarding potential ivermectin resistance in *Onchocerca* parasites, as similar resistance has already been observed in veterinary nematodes. Given urgent need to identify macrofilaricidal drugs, drug repurposing has been explored as a strategy to accelerate the development of new treatments for filariasis. This approach is not new, as several currently used antifilarial drugs including ivermectin, diethylcarbamazine, moxidectin, and doxycycline were originally developed for veterinary or other medical applications before being adapted for human use (Tagboto and Orish, 2022).

Effective vector management is crucial in controlling the spread of animal filariasis, which is transmitted by mosquitoes, black flies, and biting midges. Integrated vector management is a comprehensive strategy that utilizes multiple resources to enhance vector control. It integrates different interventions aimed at one or more diseases and employs a range of approaches, including environmental, mechanical, biological, chemical, and innovative technologies. Successful implementation depends on evidence-based decisions, coordination among sectors, strong advocacy, community participation, appropriate legislation, and capacity development (Handbook for Integrated Vector Management in the Americas, 2019). Preventing mosquito exposure is essential in minimizing the risk of filarial disease transmission. Environmental changes, including deforestation, urbanization, and irrigation, combined with socio-economic shifts, can enhance mosquito migration and dispersal via wind and water, facilitating the transmission and spread of diseases to previously non-endemic regions (Wilson et al., 2020). So, a comprehensive strategy involves environmental management, chemical control, and targeted interventions are needed. Among this, integrated mosquito management is a comprehensive approach aimed at keeping mosquito populations below levels that pose a risk to public health, thereby reducing disease transmission and, under certain conditions, achieving local elimination. This strategy is guided by four core principles. First, it emphasizes understanding the relationship between mosquito breeding patterns and environmental factors that influence their proliferation. Second, it promotes the implementation of environmental management practices to reduce or eliminate breeding sites. Third, it focuses on targeting specific mosquito species known to transmit diseases, ensuring that control efforts are species-specific and effective. Lastly, the approach prioritizes controlling mosquito population density rather than pursuing complete eradication, recognizing the ecological balance and sustainability of interventions (Zhao and Xue, 2022). Eliminating stagnant water around farms, kennels, and pastures is essential to disrupt mosquito breeding, while regular cleaning and maintenance of water troughs and drainage systems help prevent larval development. Housing livestock in well-ventilated stables equipped with insect-proof screens can significantly reduce vector entry, and the use of high-velocity fans further deters flying insects. Chemical control measures, such as applying pyrethroid-based insecticides in animal shelters and resting sites, effectively eliminate adult mosquitoes and flies. Additionally, use of larvicides like temephos and methoprene in water sources disrupts the mosquito life cycle. It has been shown that using pesticides and pour-on insecticides, like deltamethrin, is ineffective, presumably because of their brief residual activity, particularly during rainy season (Laaksonen et al., 2009). So, to maintain sufficient protection against vector exposure, its efficacy could be increased with frequent and repeated applications. Periodic fogging in high-risk areas, along with targeted insecticide spraying in cattle farms and kennels, further suppresses vector populations. Implementing these integrated control measures is essential to minimizing vector densities and reducing the risk of animal filariasis transmission. Advancing knowledge on the epidemiology, pathogenesis, diagnosis, economic impact, and zoonotic significance of animal filariasis is critical for developing effective control and prevention strategies. Implementation of the one health approach requires active collaboration among veterinary, public health, and environmental health sectors. In endemic regions, veterinary services conduct surveys for filariasis in various animal species, including dogs, cats, sheep, cattle, and horses. This integrated framework is critical for the prevention, surveillance, and control of zoonotic diseases. Without such collaboration, disease control efforts are likely to be inefficient, and the risk of outbreaks and zoonotic transmission remains high. However, routine, ongoing interdisciplinary coordination remains limited in many regions. Strengthening such collaboration through one health platforms is essential for effective prevention and control of zoonotic and vector-borne diseases.

## Conclusion

Filariasis poses a significant health threat to both humans and animals, with considerable medical, veterinary, and economic implications, affecting millions worldwide. The genetic diversity of filarial parasites can lead to varied phenotypic expressions, influencing host–parasite interactions. Effective control and elimination strategies primarily target mosquito vectors and potential reservoir hosts. The application of molecular techniques for parasite identification is crucial in developing effective strategies for managing emerging filarial infections. Comprehensive data on filariasis, including its geographic distribution, interaction with hosts and vectors, and effects on veterinary and human health, is necessary for focused interventions. Advancements in research methodologies are necessary to enhance disease detection, assess infection status, and identify more effective therapeutic targets. Currently, treatment largely depends on chemotherapy-based approaches, highlighting the need for intensified vaccine research to either prevent filarial infections or interrupt transmission. For the successful implementation of vector control measures, detailed investigations into the life cycle of filarial nematodes, particularly their developmental stages within mosquito vectors, are imperative. Understanding these key biological processes could facilitate the identification of novel intervention targets, contributing to more effective disease management strategies.

Animal filariasis, attributable to filarial nematodes such as *Setaria*, *Dirofilaria*, and *Onchocerca* species, remains a significant concern in veterinary medicine, particularly in tropical and subtropical regions. These parasitic infections not only induce considerable morbidity in domestic animals but also contribute to decreased productivity, reproductive impairments, and deterioration in hide and meat quality, collectively imposing substantial economic burdens on the livestock sector. Beyond veterinary concerns, several filarial species possess notably zoonotic potential, imposing a threat to public health and highlights the need for a one health approach to mitigate these infections. Despite being largely neglected in many parts of the world, filarial infections continue to spread due to factors such as insufficient vector control, climate change, and global animal trade. Effective control of filariasis relies on timely diagnosis, appropriate therapeutic interventions, and comprehensive management strategies encompassing vector control and prophylactic measures. However, persistent challenges in disease surveillance, diagnostic accuracy, and vaccine development continue to hinder progress in mitigating the spread of filarial infections. Addressing the burden of animal filariasis need a multidisciplinary approach involving veterinarians, public health professionals, entomologists, and researchers. Increased awareness, enhanced research funding, and the implementation of coordinated control strategies will be vital in reducing the burden of animal filariasis and preventing its spillover to human populations. The findings provide a foundation for future quantitative studies assessing infection prevalence, vector competence, and transmission dynamics.
